# Increased nitrogen supply promoted the growth of non-N-fixing woody legume species but not the growth of N-fixing *Robinia pseudoacacia*

**DOI:** 10.1038/s41598-018-35972-6

**Published:** 2018-12-17

**Authors:** Xiao Wang, Xiao Guo, Yang Yu, Han Cui, Renqing Wang, Weihua Guo

**Affiliations:** 1 Institute of Ecology and Biodiversity, School of Life Sciences, Shandong University, 27 Shanda Nanlu, Jinan, 250100 People’s Republic of China; 2 Shandong Provincial Engineering and Technology Research Center for Vegetation Ecology, Shandong University, 27 Shanda Nanlu, Jinan, 250100 People’s Republic of China; 3 College of Landscape Architecture and Forestry, Qingdao Agricultural University, 700 Changcheng Road, Qingdao, 266109 People’s Republic of China; 4 School of Life Science, Shandong Normal University, 88 Wenhua Donglu, Jinan, 250100 People’s Republic of China; 5 Institute of Environmental Research, Shandong University, 27 Shanda Nanlu, Jinan, 250100 People’s Republic of China

## Abstract

Nitrogen (N) is an essential macronutrient for plant development and growth, and the deposition of N has increased in recent decades. Legumes that fix N can also provide N for nearby species. However, N in soil inhibits N fixation. We tested the effects of N fertilisation on one N-fixing (*Robinia pseudoacacia*) and two non-N-fixing (*Sophora japonica* and *Senna surattensis*) woody legume species, which were subjected to five different N levels (0, 1.5, 2.9, 5.9 and 11.4 mg N per plant day^−1^) under greenhouse conditions. The growth of the two non-N-fixing species was promoted by N supply, while that of *R. pseudoacacia* was unaffected. Among the three species, *R. pseudoacacia* had the largest specific leaf area and chlorophyll concentration, *S. japonica* had the largest root-to-shoot ratio and main root-to-lateral root ratio, and *S. surattensis* had the largest leaf N and phosphorus concentrations. The N-fixing species was mostly unaffected by N supply. The growth, leaf chlorophyll concentration, and leaf number in the non-N-fixing species were promoted by N supply. The N-fixing species showed better growth in low-N environments, while under increased N deposition, its growth was similar to that of the non-N-fixing species.

## Introduction

Nitrogen (N) deposition has been an important part of global environmental change in recent decades^1^. Since the industrial revolution, industrial processes, agricultural development, and greater human activity have accelerated the emission of N oxides (NO_x_) and ammonia (NH_3_)^2,3^, thus increasing N deposition^4^. Nitrogen deposition in the global ecosystem was 34 Tg year^−1^ in the 1860s and 100 Tg year^−1^ in 1995 and it is projected to increase to 200 Tg year^−1^ by 2050^1,5^. Nitrogen enrichment has affected global ecosystems drastically^6,7^. Nitrogen is an essential macronutrient for plants in soil^8^ and also a key limitation to plant growth in most terrestrial forests^9^. Contrarily, excess N may lead to better growth of invasive or alien species thereby chang the biodiversity and structure of communities in particular ecosystems^6,10–12^.

Nitrogen derived from biological N fixation provides another N resource to plants^13–15^, through decomposition of the root fraction and root exudation by legumes^16^. Nitrogen fixation, however, is quite costly as compared with absorbing N from soil^17^, and can be inhibited by soil N^18^ and N deposition^19^. Legumes that can fix atmospheric N can alleviate N limitation in soil^20^. This can be seen in agricultural ecosystems, in which annual N fixation input from oilseed legumes is estimated to be 18.5 Tg^21^. Nitrogen-fixing plants can even increase the productivity of nearby plants when planted in combination with certain other species^22^. Having the ability to fix N, many legumes are less sensitive to N supply than other species^23,24^, and their photosynthetic rates are seldom affected by leaf N^25^. Therefore, the effects of N deposition on legumes are increasingly attracting the attention of ecologists^26^.

Many studies have primarily focused on herbaceous N-fixing legumes^21,27–29^, although studies on N-fixing and non-N-fixing woody legumes are also important elements in forestry and revegetation. Studies examining the differences between woody legumes under various levels of N help to clarify the effect of N supply on the N fixation process. To test the effect of N supply on N-fixing and non-N-fixing woody legume species, we chose *Robinia pseudoacacia* as the N-fixing species, and one native species, *Sophora japonica*, and one alien species, *Senna surattensis*, as the non-N-fixing species; both *S. japonica* and *S. surattensis* do not form nodules^30–32^ and cannot fix N^33^.

To test the effect of increased N supply on non-N-fixing and N-fixing legumes, five different levels of N supply were applied to seedlings of the three tree species in a greenhouse. We hypothesised that N will promote the growth of the three legume tree species, although to a lesser extent in the N-fixing species than in the non-N-fixing species.

## Results

### Plant growth

**Figure 1:**
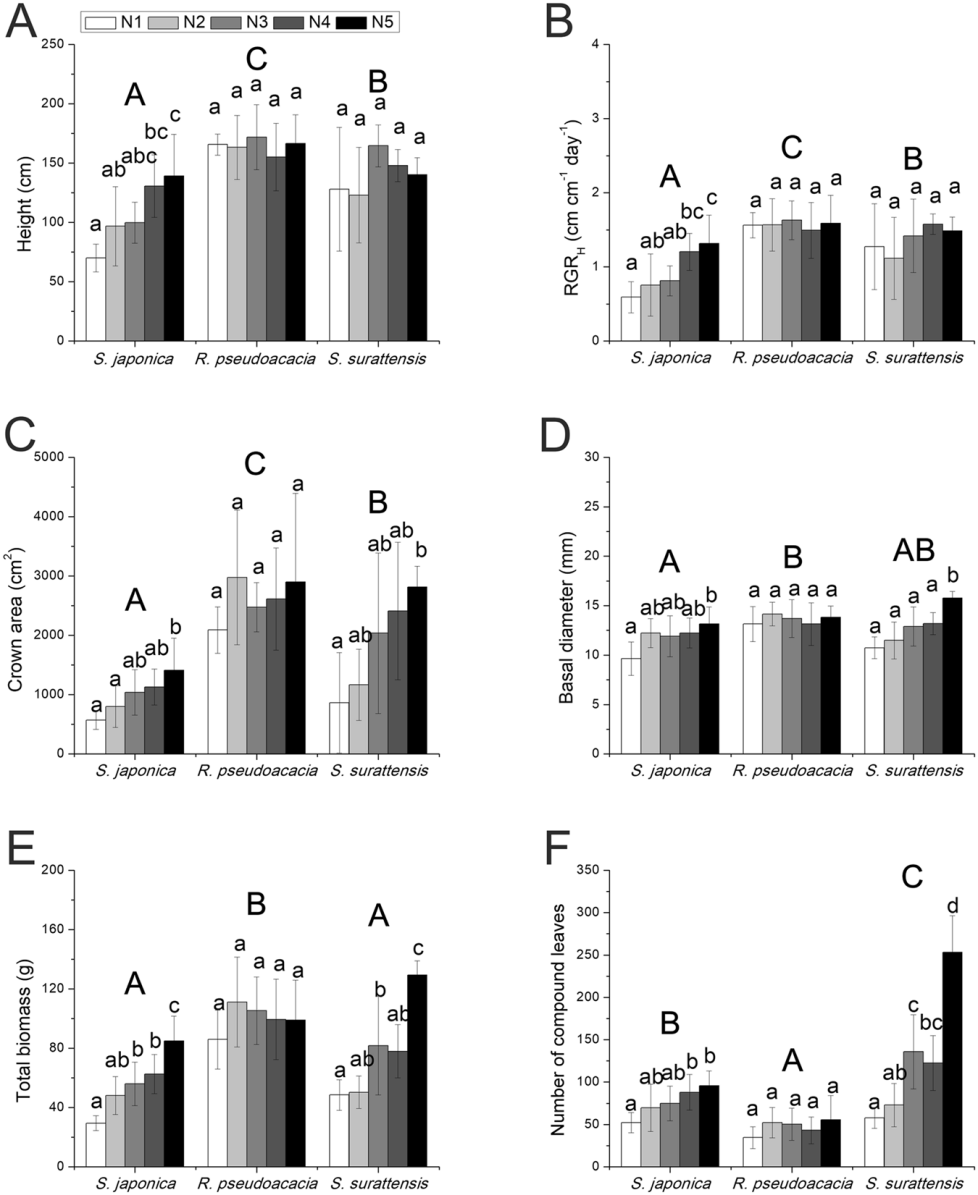
Effects of different levels of nitrogen (N) supply on plant growth in *Sophora japonica*, *Robinia pseudoacacia*, and *Senna surattensis*. (**A**) Height (n = 5–7); (**B**) Relative growth rate in height (RGRH) (n = 5–7); (**C**) Crown area (n = 5–7); (**D**) Basal diameter (n = 5–7); (**E**) Total biomass (n = 5–7); (**F**) Number of compound leaves (n = 5–7). Different lower-case letters for each species denote significant differences at various levels of N supply, and different upper-case letters denote significant differences of species under average level of N supply (p ≤ 0.05).

No significant effects of N were observed on any of the growth parameters of *R. pseudoacacia* (Fig. 1). The plant height (*p* = 0.000), relative growth rate in height (RGR_H_) (*p* = 0.000), crown area (*p* = 0.003), basal diameter (*p* = 0.010), total biomass (*p* = 0.000), and number of compound leaves (*p* = 0.004) of *S. japonica* significantly increased under an elevated nitrogen supply (Fig. 1).Increased N supply significantly increased the crown area (*p* = 0.008), basal diameter (*p* = 0.000), total biomass (*p* = 0.023), and compound leaf number (*p* = 0.000) of *S. surattensis* (Fig. 1).

*Sophora japonica* had the lowest height (*p* = 0.000), RGR_H_ (*p* = 0.000), crown area (*p* = 0.000) and basal diameter (*p* = 0.001), while *R. pseudoacacia* had the greatest height, RGR_H_, crown area, basal diameter and total biomass (*p* = 0.000), but the least number of compound leaves (*p* = 0.000) among the three species under the average N supply (Fig. 1).

### Biomass allocation

**Figure 2:**
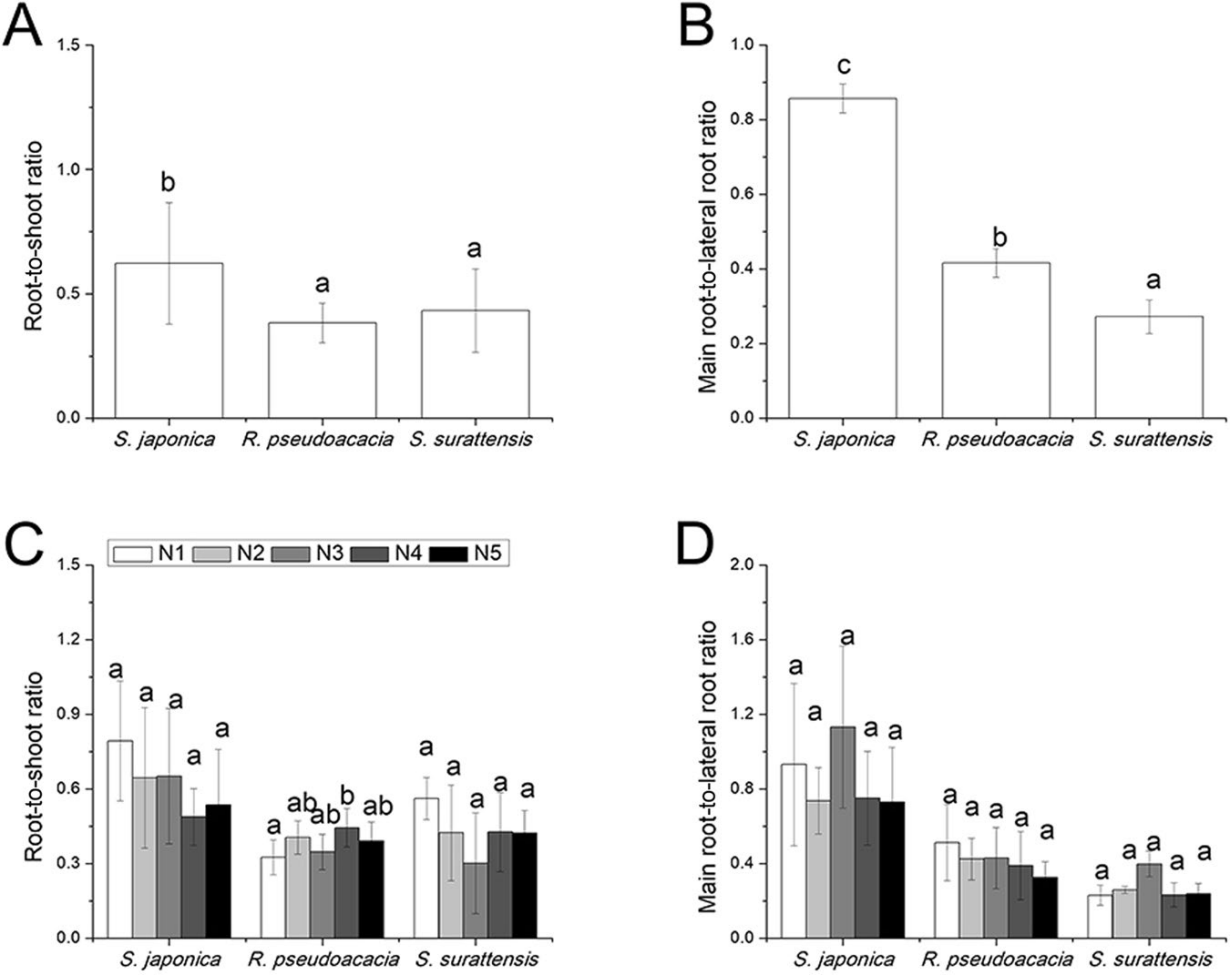
Difference in biomass allocation (**A**, **B**) and the effect of nitrogen on biomass allocation (**C**, **D**) in *Sophora japonica*, *Robinia pseudoacacia*, and *Senna surattensis*. (**A**) Root-to-shoot ratio (n = 27–35); (**B**) Main root-to-lateral root ratio (n = 27–35); (**C**) Root-to-shoot ratio (n = 5–7); (**D**) Main root-to-lateral root ratio (n = 5–7). Different letters for each species denote significant differences (*p* ≤ 0.05).

Nitrogen treatment did not significantly affect the root-to-shoot ratio and main root-to-lateral root ratio of any of the three species (Fig. 2C,D). *Sophora japonica* had higher root-to-shoot ratio than the other two species (*p* = 0.000) (Fig. 2A). The main root-to-lateral root ratio was the highest in *S. japonica* and the lowest in *S. surattensis* (*p* = 0.000) (Fig. 2B).

### Leaf traits

**Figure 3:**
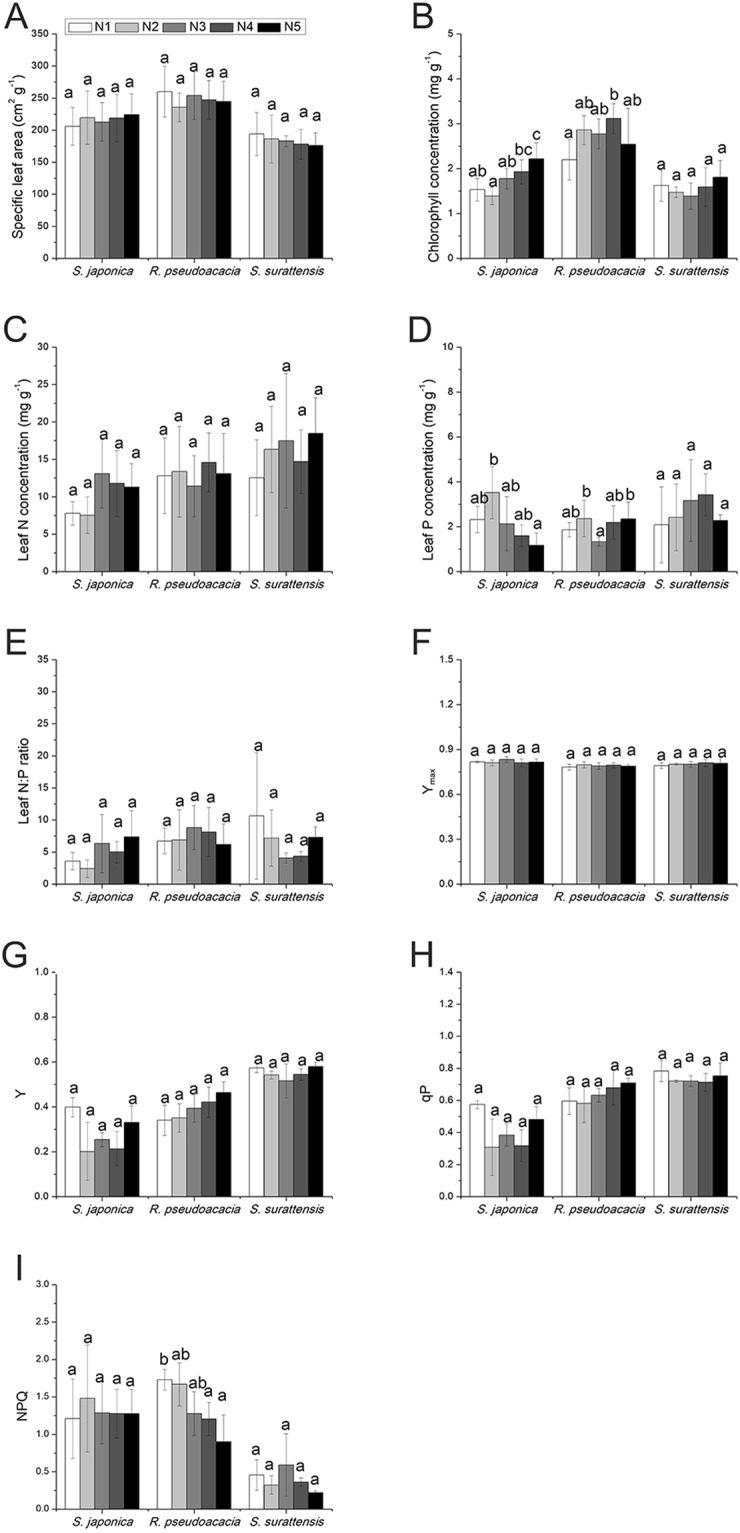
Effects of different levels of nitrogen deposition on leaf traits in *Sophora japonica*, *Robinia pseudoacacia*, and *Senna surattensis*. (**A**) Specific leaf area (n = 5–7); (**B**) Chlorophyll concentration (n = 5–7); (**C**) Leaf nitrogen (N) concentration (n = 5–7); (**D**) Leaf phosphorus (P) concentration (n = 5–7); (**E**) Leaf nitrogen to phosphorus (N:P) ratio (n = 5–7); (**F**) Maximum quantum yield (Y_max_) (n = 5–7); (**G**) Effective quantum yield (Y) (n = 3); (**H**) Photochemical quenching (qP) (n = 3); (**I**) Non-photochemical quenching (NPQ) (n = 3). Different letters for each species denote significant differences (*p* ≤ 0.05).

Chlorophyll concentration in *S. japonica* significantly increased with increasing N supply (*p* = 0.000), while leaf phosphorus (P) concentration significantly decreased under elevated N levels (*p* = 0.005) (Fig. 3). Chlorophyll concentration in *R. pseudoacacia* also significantly increased with the addition of more N (*p* = 0.016), while non-photochemical quenching (NPQ) decreased (*p* = 0.020) (Fig. 3). The leaf P concentration in *R. pseudoacacia* was also slightly affected by N. None of the leaf traits of *S. surattensis* were significantly affected by N (Fig. 3).

**Figure 4:**
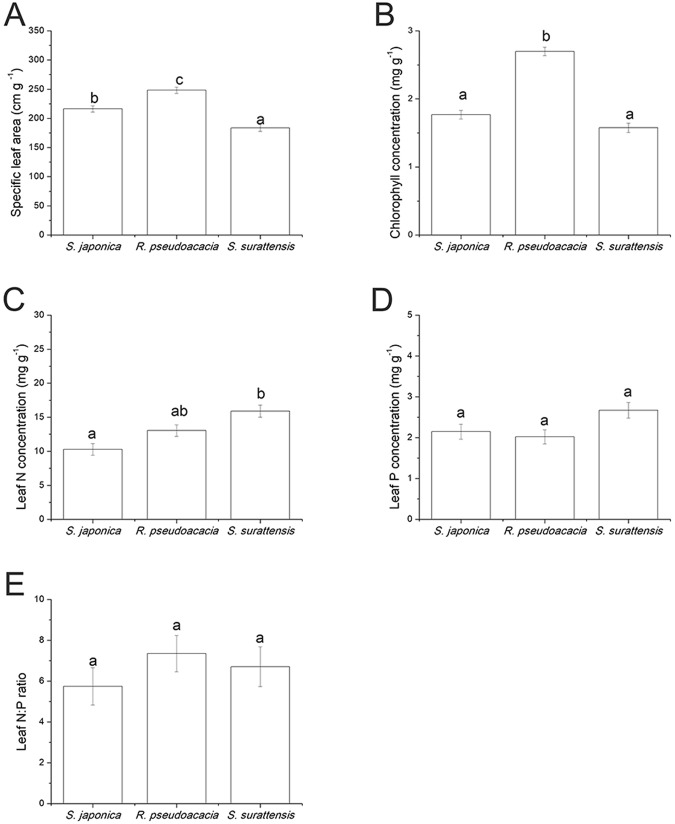
Differences in leaf traits of *Sophora japonica*, *Robinia pseudoacacia*, and *Senna surattensis*. (**A**) Specific leaf area (n = 27–35); (**B**) Chlorophyll concentration (n = 27–35); (**C**) Leaf nitrogen (N) concentration (n = 27–35); (**D**) Leaf phosphorus (P) concentration (n = 27–35); (**E**) Leaf nitrogen to phosphorus (N:P) ratio (n = 27–35). Different letters for each species denote significant differences (*p* ≤ 0.05).

Among the three species, *R. pseudoacacia* had the highest specific leaf area (*p* = 0.000), while *S. surattensis* had the lowest specific leaf area (Fig. 4A). *Robinia pseudoacacia* had higher chlorophyll concentration than the other two species (*p* = 0.000) (Fig. 4B). *Senna surattensis* had the highest leaf N (*p* = 0.000) and relatively high P concentration, while *S. japonica* and *R. pseudoacacia* had the lowest N concentrations (Fig. 4C,D). Leaf N:P ratios of all three species were similar (Fig. 4E).

## Discussion

Increased N supply promoted the growth of *S. japonica* and *S. surattensis*, but not the growth of *R. pseudoacacia*. During the entire experimental period no root nodules were found on plants of *S. japonica* and *S. surattensis* while *R. pseudoacacia* had root nodules under all N levels. Given that legumes cannot fix nitrogen without root nodules^33^, our data confirm that *R. pseudoacacia* was the only of the three species that fixed N. Previous studies have reported that growth of non-N-fixing plants is mostly promoted by elevated N supply^34–36^. However, in species that can fix N (e.g., soybean or lupin), growth is often less or even not affected by N fertilization^37,38^; this was confirmed in our study. Previous studies have shown that N fertiliser can inhibit N fixation^18,39,40^, which explains a similar growth of *R. pseudoacacia* at both high and low N supplies. The growth of *R. pseudoacacia* under low soil N levels was not limited because the plant was able to fix the required N from the atmosphere.

Nitrogen did not affect biomass allocation, leaf N concentration, or leaf N:P ratio of the three species. Biomass allocation is affected by N in many plant species^34,41–44^, but it is independent of N in many N-fixing legumes^24^. In our study, N did not affect biomass allocation in the three species, suggesting that this trait may be unrelated to N fixation and is specific to legumes. However, this remains to be tested in other N-fixing and non-N-fixing species from this plant group. Increased chlorophyll concentrations in *S. japonica* and *R. pseudoacacia* at higher N levels suggests higher photosynthetic rate^45,46^. Leaf N concentrations were not affected by soil N in our experiment, it has been reported that leaf N is unrelated to photosynthesis in most N-fixing legumes^25^, and this may also be the same in non-N-fixing legumes. Leaf P concentration in *S. japonica* decreased under higher N levels and was the lowest of the three species, suggesting that the ability of *S. japonica* for absorbing P from soil was weaker than that in the other two species. This was also evident from the main root-to-lateral root ratio, which was the lowest in *S. japonica* owing to its lower nutrient exploiting efficiency when compared with the other two species^47^.

The similar growth of *R. pseudoacacia* at different N levels is related to photosynthesis and carbon cost. It has long been reported that under low N conditions plants tend to have a higher photochemical efficiency of the photosystem II (PSII) and photochemical quenching (qP), but lower NPQ^48–52^. In the present study, higher N levels decreased the NPQ, but N supply had no effect on qP and PSII photochemical efficiency. This absence of correlation between N levels and PSII photochemical efficiency and higher chlorophyll concentrations suggested that the photosynthetic rate in *R. pseudoacacia* was similar at high and low N levels. This similar photosynthetic rate at different N levels may be attributed to the presence of reactive oxygen species. Namely, qP and NPQ may contribute to the decreased formation of singlet oxygen, a type of reactive oxygen species^53^. Therefore, similar qP and a decreased NPQ under high N levels may indicate increased production of singlet oxygen, which can damage PSII^54^. At low N supply, *R. pseudoacacia* may have higher N fixation rate thus expending more energy and carbon^17^, while at higher N levels, the plants may have similar or higher photosynthetic rates and may also produce more singlet oxygen that can damage PSII. Thus, despite having higher chlorophyll concentration, the photosynthetic rate in *R. pseudoacacia* at higher N levels, owing to PSII damage, may be similar or even lower than that in plants at low N levels^55^.

Different source activities were observed in the three species. Nitrogen has increased sink capacity^56^, which in turn, promotes source strength^57,58^. *Sophora japonica* at high N levels tended to increase the strength of the source (photosynthetic rate) and the number of sources (number of compound leaves) to get a higher relative growth rate and biomass accumulation. *Robinia pseudoacacia* under high N levels seemed to increase the strength of the source although incurring PSII damage. *Senna surattensis* at higher N levels increased only the number of sources to enhance their growth.

The difference among species and the varied response to N may be related to different ecological strategies of plant species. *Robinia pseudoacacia*, which was dominant under low N conditions in our experiment, has the ability to fix N^59^. Its higher biomass use efficiency in leaves, when compared with other species, was reflected in its large specific leaf area, and its predicted high photosynthetic rate (high chlorophyll concentration)^45,46^, as well as its ability to fix N, make it a more invasive species that can adapt to different soil types^60^. Invasion by *R. pseudoacacia* may lead to an increase of soil N thus may cause biodiversity loss^61^. The relatively low leaf P concentration in *R. pseudoacacia* may be due to the strong sink of P in its nodules^62,63^. *Senna surattensis* had the lowest main root to lateral root ratio, which shows that its roots have a higher exploitation efficiency^47^. The N and P concentration was relatively high in the leaves of *S. surattensis*, which also demonstrated its high absorption rate of nutrients from the soil^64^. This was confirmed as *S. surattensis* is mainly distributed in South Asia^65^, where lower soil P concentrations are observed^66^. Unlike *R. pseudoacacia*, which is an early successional species with high foliar efficiency, *S. surattensis* and *S. japonica* are more likely to be late successional species^67^. *Sophora japonica* in our study was never dominant under any N conditions; it is also observed to be non-dominant in China, especially the northern regions^68^. Its large main root-to-lateral root ratio revealed its low exploitation efficiency of nutrients from the soil. This may explain its distribution in northern China, where there are higher nutrient levels in the soil^66^. Studies at the molecular level are still needed to quantify the difference of N-fixing and non-N-fixing legumes.

Our study showed that the growth of the N-fixing species *Robinia pseudoacacia* was not affected by different levels of N supply. In contrast, the growth of the non-N-fixing legume species was promoted by increased N supply. Similar growth in both the non-N-fixing species and *R. pseudoacacia* was observed under high N supply. Matured leaves, which are the source part of plants, increased in number in non-N-fixing species under increased N supply. The leaf number in *R. pseudoacacia* as well as total biomass, were similar under all N levels. Biomass allocation and leaf N, that were not affected by N supply, may have been legume-specific properties, rather than being specific to N-fixing species. The traits’ difference between species may reveal a different adaptation ability, which might affect their distribution in the future, characterised by increased N supply. The non-N-fixing species grew better under high N supply conditions than under low N supply, which provides evidence for planted forestry and garden greening when choosing woody species and managing fertilisation. Further studies are required on the effect of N on matured woody legumes.

## Materials and Methods

### Study site

Our experiments were conducted at the Fanggan Research Station of Shandong University (36°26′ N, 117°27′E) in Laiwu, Shandong Province, China. The study site is characterized by warm temperate monsoon climate, with a mean annual temperature of 13 ± 1 °C and a mean annual precipitation of 700 ± 100 mm. Most of the rainfall is concentrated in summer and early autumn^69^. The entire experiment was carried out in a greenhouse during the growing season.

Seeds of *R. pseudoacacia*, *S. japonica*, and *S. surattensis* were germinated in late April. Subsequently 35 healthy, similar-sized seedlings of each species were transplanted into pots filled with 6 kg of loam and 2 kg of sand on May 5 (one plant per pot for each species). The substrates, which were thoroughly mixed, had the following chemical properties: 50.20 mg kg^−1^ available N, 31.14 mg kg^−1^ available P, and pH 6.51. The plants were watered every 2 days. After being transplanted into pots, the plants were treated with different amounts of N on June 15^th^.

### Experimental design

Each species was treated with five N levels: 0, 1.5, 2.9, 5.9 and 11.4 mg N per plant day^−1^ (N1, N2, N3, N4, and N5, respectively). Ammonium nitrate (NH_4_NO_3_) was used as the source of N. Nitrogen application began on June 15^th^ and the NH_4_NO_3_ solution was added to the pots five times at 15-day intervals. No inoculation was performed, and root nodules were formed with natural rhizobia present in the unsterilized soil. All the plants were harvested on September 10^th^, i.e. the second week after the last N application. There were seven replicates of each treatment for each species. All pots were randomly arranged in the greenhouse. All plants were watered every 2 days during the experiment.

### Measurements

Seedling height and crown area (calculated as crown area = 0.5*ab*, where *a* and *b* are the vertical diagonals of the crown area) were measured at the beginning and the end of N treatments. The relative growth rate in height (RGR_H_) was calculated with the formula: RGR_H_ = (ln*H2*−ln*H1*)/*t*, where *H2* and *H1* are the seedling height at the end and the beginning of the N treatment, respectively, and *t* is the duration of the experiment. The basal diameter was measured before harvest. Flower number (if present) and leaf number were counted at harvest time. Before harvest, the fourth mature leaf from the top was sampled to determine the specific leaf area of plants using the formula: specific leaf area = leaf area/leaf dried biomass, where leaf area was calculated by WinFOLIA Pro 2009a software (Regent Instruments, Inc., Quebec, Canada). Before harvest, the fifth fully expanded mature leaf from the top was collected to measure chlorophyll concentration, using a spectrophotometric method^70^. Chlorophyll fluorescence parameters were measured using a pulse amplitude modulation chlorophyll fluorometer (Mini-PAM; Walz GmbH, Effeltrich, Germany). The parameters were measured as follows: maximum quantum yield of PSII in dark-adapted leaves, *Y*_*max*_ = (*F*_*m*_ − *F*_*o*_)/*F*_*m*_; effective quantum yield of PSII, *Y* = (*F*_*m*_′ − *F*)/*F*_*m*_′; photochemical quenching, qP = (*F*_*m*_′ − *F*)/(*F*_*m*_′ − *F*_*o*_′); and non-photochemical quenching, NPQ = (*F*_*m*_ − *F*_*m*_′)/*F*_*m*_′, where *F*_m_ and *F*_m_′ are maximum fluorescence and peak value of fluorescence; *F*_*o*_ is minimum fluorescence after dark adaptation and *F*_*o*_′ = *F*_*o*_/((*F*_*m*_ − *F*_*o*_)/*F*_*m*_* + F*_*o*_/*F*_*m*_′). During harvest, compound leaf numbers of each plant were recorded, plants were cut at ground level, and the underground parts were carefully washed with tap water. All plants were separated into five parts: main root, lateral root, stem, leaf blade, and leaf petiole. The parts of each plant were packed in separate envelopes and oven dried for 48 h at 80 °C. Once the material was completely dried, all parts were weighed. Plant biomass allocation was calculated, based on dried biomass, as follows: total biomass = main root biomass + lateral root biomass + leaf blade biomass + petiole biomass + stem biomass; root to shoot ratio = (main root biomass + lateral root biomass)/(leaf blade biomass + petiole biomass + stem biomass); main root to lateral root ratio = main root biomass/lateral root biomass. Leaf N and P concentrations were measured using the Kjeldahl method^71^ and colorimetric determination^72^, respectively.

### Statistical analyses

The data were analysed using one-way analysis of variance (ANOVA) and Tukey’s tests at *p* ≤ 0.05. Data were checked for normality and homogeneity of variance before performing ANOVA. Log transformation was applied when data did not meet the criteria for normality and homogeneity. Transformed data that still did not meet the criteria for normality and homogeneity of variance were analysed by non-parametric tests. All the data were analysed using SPSS 19.0 software (SPSS Inc., Chicago, IL, USA). Figures were drawn using Origin 8.0 software (OriginLab Co., Northampton, MA, USA).

## Data Availability

The datasets generated during and/or analysed during the current study are available from the corresponding author on reasonable request.
